# Impact of cardiac rehabilitation and treatment compliance after ST-segment elevation myocardial infarction (STEMI) in France, the STOP SCA+ study

**DOI:** 10.3389/fcvm.2025.1484401

**Published:** 2025-06-12

**Authors:** Emeline Laurent, Lucile Godillon, Marc-Florent Tassi, Pierre Marcollet, Stéphan Chassaing, Marie Decomis, Julien Bezin, Christophe Laure, Denis Angoulvant, Grégoire Range, Leslie Grammatico-Guillon

**Affiliations:** ^1^ Public Health Unit, Epidemiology, Teaching Hospital of Tours, Tours, France; ^2^ Research Unit EA 7505 “Education, Ethics and Health”, University of Tours, Tours, France; ^3^ Faculty of Pharmacy, University of Tours, Tours, France; ^4^ Cardiology Department, CH Bourges, Bourges, France; ^5^ Cardiology Department, Private Hospital NCT+, Tours, France; ^6^ Cardiology Department, Private Hospital Oréliance, Orléans, France; ^7^ Clinical Pharmacology Unit, University of Bordeaux, INSERM, BPH, Team AHeaD, Bordeaux, France; ^8^ Cardiology Department, Les Hôpitaux de Chartres, Chartres, France; ^9^ Cardiology Department, Teaching Hospital of Tours, Tours, France; ^10^ Faculty of Medicine, University of Tours, Tours, France

**Keywords:** myocardial infarction (MI), cardiac rehabilitation, compliance, outcome, probabilistic matching

## Abstract

**Introduction:**

Acute ST-elevation myocardial infarction (STEMI) is a frequent and serious presentation of acute coronary syndrome. The STOP-SCA+ study aimed to (i) describe 1-year compliance to secondary prevention cardiac tri-therapy and (ii) identify factors associated with negative outcomes 1 year after STEMI, particularly the impact of compliance and rehabilitation care.

**Methods:**

Patients who were >18 years old and hospitalized for STEMI in five interventional cardiac centers with the same cardiac registry in one French region (2.5 million inhabitants), between 2014 and 2018, were included. After a probabilistic matching with the National Health Insurance database [Système National des Données de Santé (SNDS), 96% matching], compliance for cardiac tri-therapy was studied: aspirin, P2Y12 inhibitor, and statin. Factors associated with poor outcomes (ischemic complications, death) were analyzed using Cox modeling and those for compliance by logistic regression.

**Results:**

A total of 3,768 patients were included, of whom 84% underwent primary percutaneous coronary intervention. At 1 year, 3,362 had at least one tri-therapy delivery (89.2%), of whom 53% were compliant, and 2,478 patients went to cardiac rehabilitation (65.8%). Death occurred in 130 patients and/or ischemic complications in 194 (total of poor outcomes 8.0%). Compliance was not associated with complications over the year [HR 1.16 (0.86–1.57)], while the absence of cardiac rehabilitation [2.31 (1.73–3.08)] was associated, as well as female sex 1.54 (1.08–2.19), renal impairment [2.87 (1.49–5.53)], initial STEMI clinical presentation [pejorative Killip 2.04 (1.19–3.50)], and LVEF <40% at discharge [2.22 (1.65–2.99)]. Additionally, cardiac rehabilitation was associated with compliance [OR 1.55 (1.34–1.79)].

**Discussion:**

Pejorative outcomes 1 year after a STEMI represented 8% of cases, mainly related to patient features, the initial clinical presentation, and the absence of access to rehabilitation. Compliance part in patient health outcomes will need further modeling to accurately study its impact. Matching clinical and medico-administrative databases proved to be relevant for assessing outcomes at a large scale.

## Introduction

Cardiovascular diseases are a major cause of death in France, mainly due to coronary artery disease and its major complications, i.e., acute coronary syndrome (1–3). Acute ST-elevation myocardial infarction (STEMI) is a frequent and serious presentation (4–6). The reference treatment for STEMI consists of performing timely revascularization by either primary angioplasty (percutaneous intervention PCI) or pharmacological fibrinolysis. PCI is the preferred alternative if it can be performed in <120 min between diagnosis and the interventional procedure (3, 7–9), to shorten the myocardial ischemia and improve the functional recovery and the survival rate (8, 10–12). The prescription and compliance with a cardiac treatment for tertiary prevention are also key factors for improving the STEMI prognosis (4). Indeed, drug management after myocardial infarction (BASI: Beta-blocker, Antiplatelet therapy, Statin, Angiotensin-converting enzyme Inhibitor) must be optimized and maintained over time to prevent ischemic recurrences and death, with a high-level recommendation for a dual antiplatelet therapy DAPT (including aspirin and P2Y12 inhibitor) in the following 1-year period after PCI, along with a long-term LDL cholesterol-lowering therapy (4, 13). However, little is known about compliance to secondary prevention cardiac tri-therapy at 1 year, due to difficulties in real-life assessment, as well as about the factors associated with this compliance and, eventually, the impact of a lack of compliance.

Using an automated procedure to monitor the immediate outcomes of STEMI patients and their follow-up, including medication intake, could provide high-quality data without significant cost and time waste (14). Therefore, in the French region of Centre-Val de Loire [approximately 2.5 million inhabitants and six interventional cardiology centers (ICC)], an electronic registry was established in 2014 (CRAC registry). This registry is fully integrated with the coronary activity report software and has automatic daily updates (3, 8). The CRAC registry has already provided insights into several factors that impact STEMI mortality, such as patient age, Killip score at admission, or the positive impact of calling the French medical emergency number (3, 9). However, some registry variables related to the 1-year follow-up, particularly therapeutic compliance, are collected by phone calls, which can sometimes be incomplete (not collected for 13% of the patients due to lost to follow-up/death). These missing data could be collected using the National Health Insurance database [*Système National des Données de Santé* (SNDS)].

The main objective of the cohort study, STOP-SCA+, was to describe compliance to secondary prevention cardiac tri-therapy (i.e., aspirin, P2Y12 inhibitor, and statin) of patients over the 1-year period after STEMI, by matching the CRAC registry with the SNDS. The secondary objectives were to identify (i) the factors associated with poor outcomes (death or ischemic complications) at 1 year, especially the impact of rehabilitation care and cardiac tri-therapy compliance, and (ii) the factors associated with compliance to the secondary prevention cardiac tri-therapy.

## Methods

This study was a retrospective, multicenter, and observational cohort study with a probabilistic matching (in the absence of a common direct identifier for each patient) between two databases, the CRAC registry and the medico-administrative SNDS database.

Eligible patients were all patients aged 18 years or older, diagnosed with STEMI, who underwent coronary angiography or PCI within 24 h of symptom onset, in five French ICCs, between 1 January 2014 and 31 December 2018, as identified from the CRAC registry (3). Exclusion criteria were the absence of consent, death occurring during the inclusion ICC hospital stay, or the absence of matching between the CRAC registry and SNDS data.

### Data sources and matching

The CRAC registry includes up to 150 variables for each patient, including pre-admission, hospital, and follow-up data, as described elsewhere (3). Among the 4,179 patients included in the CRAC registry over the study period, only 147 were lost to follow-up at 1 year (3.5%), underlying the quality of the monitoring and CRAC registry.

The SNDS includes the vital status and all reimbursement data for out-of-hospital drug prescriptions and consultations, along with all hospitalizations in public and private sectors in France (although without detail about drug consumptions), linked by a unique encrypted patient number (14, 15). The probabilistic matching between the CRAC registry and the SNDS was performed as described in Supplementary Tables S1.1 and S1.2. Each of the seven matching steps had excellent performance parameters, with predictive positive values reaching >99.9% for each step (the lowest parameter performance was a sensitivity estimation at 82% for one step involving only a few cases). Overall, 96% of the CRAC registry cases could be matched with their counterpart SNDS unique cases (Supplementary Figure S1.3).

This study was compliant with French regulations about data protection and authorized by the French Data Protection Board [*Commission Nationale de l'Informatique et des Libertés* (CNIL)], decision DR-2021-025 (28 January 2021), as required by French regulation (16). This study required neither information nor consent of the individuals included.

### Main outcome and variables of interest

The main outcome was defined as the occurrence of an ischemic complication or death during the first year after being discharged alive from the ICC. The complications were extracted from the CRAC registry, both (i) ischemic (stent thrombosis, myocardial infarction, non-hemorrhagic stroke, unplanned revascularisation, target lesion revascularisation) and (ii) hemorrhagic (hemorrhagic stroke, severe bleeding; BARC ≥3). The vital status was checked using both the CRAC registry and SNDS data up to 1 year after discharge. For compliance assessment, three therapeutic classes were considered: aspirin, P2Y12 inhibitors, and statin. These medications were considered separately or combined as (i) DAPT (aspirin + P2Y12 inhibitor) or (ii) tri-therapy: DAPT + statin. Patients with one antiplatelet agent (aspirin or P2Y12 inhibitor) associated with an anticoagulant were assimilated to DAPT patients. This association allowed us to consider patients with a contraindication to aspirin. For each medication, the SNDS database provides the delivery dates, which were used as proxies for administration dates, and the number of boxes and tablets dispensed in an out-of-hospital pharmacy after discharge (duration prescribed not known). Compliance with tri-therapy was the main variable of interest and was defined in two different ways. First, as a patient variable, by the percentage of days covered (PDC) by drug deliveries up to 1 year (or up to death before 1 year). For this analysis, good compliance was defined by a PDC ≥80% (17–19). Second, compliance was also defined as a time-dependent variable, with a period of exposure defined as a period covered by delivery of all three medications, whereas a non-exposure period corresponded to a period of at least 1 day when at least one medication was missing, out of three. Each hospitalization period, both in acute or rehabilitation care, was considered a full-exposure period for deliveries registered before or after the hospitalization, as hospital deliveries are not reported in the SNDS. For cardiac rehabilitation, all hospitalizations with at least one diagnosis code Z50.0 as main care were considered, including outpatient stays.

The other variables of interest included sociodemographic characteristics [age, sex, social deprivation according to the French deprivation index “FDep” for each French area (20), divided into five quintiles], underlying conditions (such as hypertension and diabetes mellitus), medical history (such as myocardial infarction and stroke), clinical presentation at onset (including the Killip score), procedural data, left ventricular ejection fraction (LVEF) at discharge, and hemorrhagic complications over the year after discharge, extracted from the CRAC registry. Cardiac rehabilitation and general practitioner (GP) consultations were extracted from the SNDS over the year after discharge. The reasons for the absence of admission to cardiac rehabilitation are not available in the SNDS. Rehospitalizations were studied and defined as acute care hospitalizations with at least one night, occurring after discharge from the index stay. Other cardiac drugs delivered (beta-blockers, angiotensin-converting enzyme inhibitors) could not be analyzed in the STOP-SCA+ study, due to variations in dosage and posology that could not be tracked via the SNDS.

### Statistical analyses

First, patients' features, clinical presentations, and 1-year outcomes were described in terms of numbers and percentages/frequencies. Median times between discharge from STEMI hospital stay and admission to a rehabilitation center were calculated, along with their first (Q1) and third (Q3) quartiles. Compliance was described at a patient level using the PDC.

Second, the factors associated with the occurrence of an ischemic complication or death were identified, with (i) log-rank tests in univariable analyses and then (ii) Cox modeling in multivariable analysis, including tri-therapy as a time-dependent variable. Cardiac rehabilitation was also included as a time-dependant variable in this model, to control a potential immortal time bias. For the final model (see below), the absence of interaction between cardiac rehabilitation and compliance was checked. Hazard ratios (HR), along with their 95% confidence interval (95% CI), were reported.

We also performed sensitivity analyses to study the association between cardiac tri-therapy and complication/death occurrence (i) by adjustment on a propensity score (ii) and by inverse probability of treatment weighting (IPTW).

Third, after checking the absence of interaction between cardiac rehabilitation and compliance in the previous model, a secondary analysis was performed to assess factors associated with compliance (defined as the proportion of days covered PDC ≥80%) for cardiac tri-therapy in a subgroup of patients without major adverse cardiac or cerebral events during the subsequent year. After a univariable analysis using Chi-square tests, a multivariable logistic regression model was performed, giving odds ratio (OR), along with their 95% CIs.

For all multivariable analyses, all variables with *p* < 0.2 in univariable analysis were included in the initial model, the final model being selected through a descending stepwise process, to select variables with *p* < 0.05, along with clinically pertinent variables (as defined by cardiologists), sex, and age. For all tests, the threshold for statistical significance was set to 5%. All analyses were conducted using SAS Enterprise Guide software (SAS Institute Inc., Cary, NC, USA), a version available on the national SNDS portal at the time of the analyses.

## Results

From the 4,179 eligible patients from the CRAC registry, 3,768 were included (90% of the eligible patients) and discharged alive with available SNDS data over the follow-up year (Supplementary Figure S1.3). The mean age was 62 years old (min–max 18–96), with three times more men than women (Table 1). Concerning the clinical presentation at onset, 3.6% had a Killip at III or IV, the main localization was inferior ischemia (53.7%), and the pre-PCI TIMI flow was grade 0–1 in almost 61% of cases.

**Table 1:** Patients’ characteristics and hospital management—the STOP-SCA+ study.

The STOP-SCA+ study: patients' characteristics	Patients included		
	*n*	% (in line)	Missing
Total	3,768	*100*	
Age, years [mean (min–max)]	62 (18–96)		0
Women	936	24.8	0
Comorbidities			
Overweight (25 ≤ BMI < 30 kg/m^2^)	1,599	*42*.*5*	4
Obesity (BMI ≥ 30 kg/m^2^)	789	*21*.*0*	4
Renal impairment	52	*1*.*4*	36
High blood pressure	1,510	*40*.*3*	25
Diabetes	519	*13*.*9*	25
Smoking (current or past)	2,006	*53*.*5*	18
Medical history			
TCA/myocardial infarction/coronary bypass	465	*12*.*3*	0
Stroke	94	*2*.*5*	3
Peripheral vascular disease	99	*2*.*6*	10
Family history of coronary disease	797	*21*.*8*	115
Killip at the admission			72
III	52	*1*.*4*	
IV	80	*2*.*2*	
Ischemia location			31
Inferior	2,006	*53*.*7*	
Anterior	1,448	*38*.*7*	
Lateral	268	*7*.*2*	
Circumferential	15	*0*.*4*	
Reperfusion procedure	3,497	*92*.*8*	0
Primary angioplasty	3,171	*84*.*2*	
Isolated fibrinolysis	54	*1*.*4*	
Angioplasty after fibrinolysis	272	*7*.*2*	
Isolated coronary angiography	271	*7*.*2*	
Pre-PCI TIMI flow grade 0–1	2,246	*60*.*8*	71
Post-PCI TIMI flow grade 0–1 or no reflow	77	*2*.*4*	519*
Median time (min)			
From symptom onset to ECG	100		803**
From ECG to primary angioplasty	101		653**
From ECG to fibrinolysis	20		73**
LVEF at discharge < 40%	665	*19*.*5*	362

The italic values in % (percentage in line): number of patients with the characteristic divided by the total number of patients included.

BMI, body mass index; TCA, transluminal coronary angioplasty; PCI, percutaneous coronary intervention; LVEF, left ventricular ejection fraction.

* Including patients without any PCI (*n* = 263).

** Before 2015 median time not measured and counted as missing data.

More than half of the patients were smokers (53%), 40% had hypertension, and 22% reported a family history of coronary disease. Obesity was observed in 21% of the cases, and diabetes mellitus was present in 14%. Among the cohort, 93% underwent revascularization (84% had a primary angioplasty). At discharge, 20% of the patients had an LVEF <40% (Table 1).

**Figure 1:**
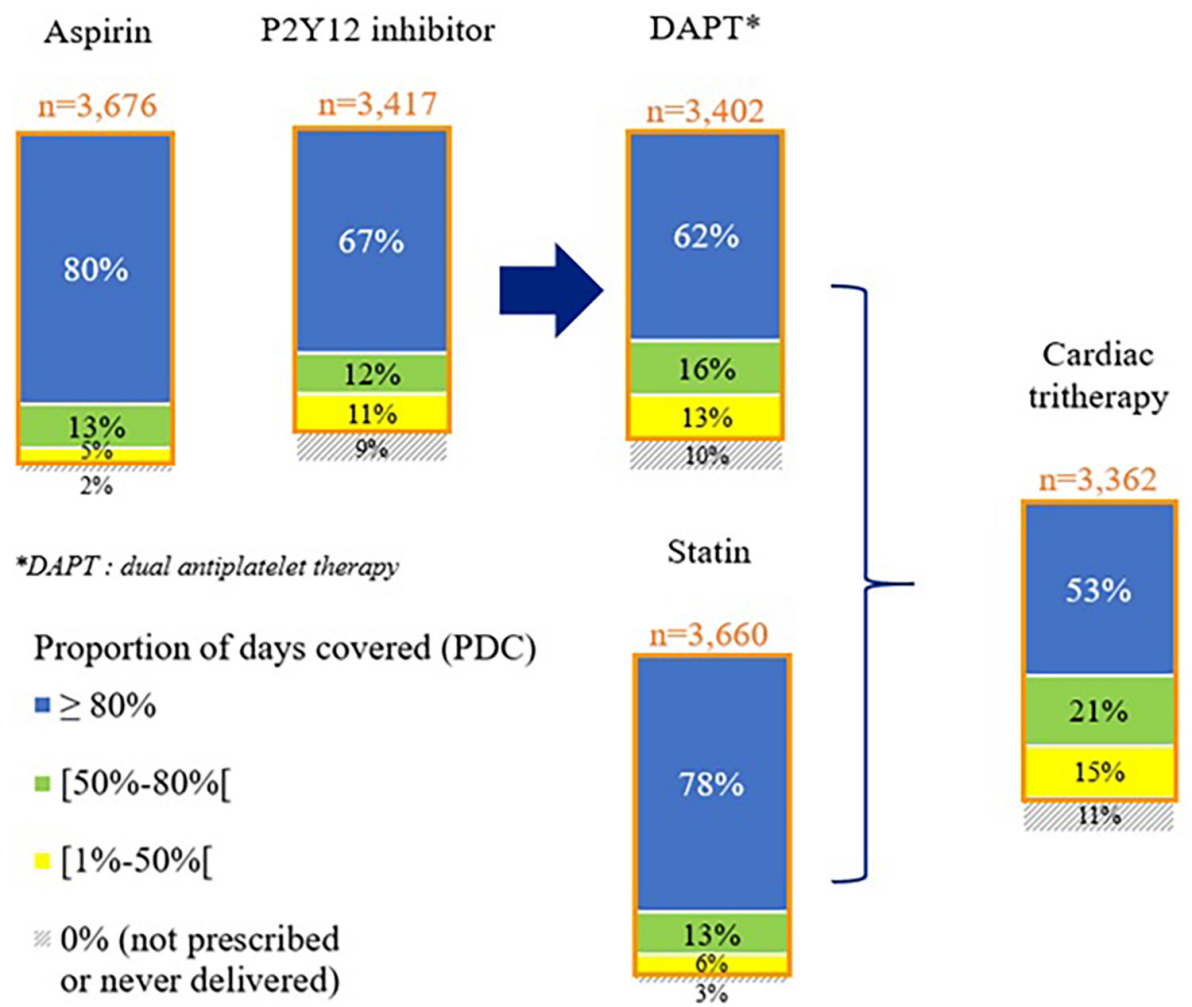
Cardiac drug compliance at 1 year after STEMI—the STOP-SCA+ study. PDC, proportion of days covered by the studied medication.

During the 1-year follow-up, 98% of the patients were prescribed at least once aspirin, 90% a P2Y12 inhibitor, and 97% a statin, resulting in 89% of the patients with at least one delivery of a cardiac tri-therapy (*n* = 3,362). Among these patients, 53% were compliant with their tri-therapy (PDC ≥80%) (Figure 1, Supplementary Figures S2, S3, and S4).

At 1 year, 66% of the STEMI patients had access to a cardiac rehabilitation center (Table 2). The median time between the STEMI patient discharge from the acute care setting to admission to a cardiac rehabilitation center was 8 days (Q1, 1 day; Q3, 20 days). The median time between the reperfusion and the admission to a cardiac rehabilitation center was 14 days (Q1, 8 days; Q3, 26 days). These median times were not different between the patients with or without complications.

**Table 2:** Patients’ outcome at 1 year after STEMI—the STOP-SCA+ study.

The STOP-SCA+ study: 1-year outcome	Patients included	
	*n*	%
Total	3,768	*100*
One-year rehospitalizations		
Acute care (full hospitalization)	1,817	*48*.*2*
With vascular disease	1,327	*35*.*2*
With emergency admission for vascular disease	299	*7*.*9*
Rehabilitation care		
In-hospital stay (≥1 night)	1,787	*47*.*4*
One-day hospitalization (0 night)	994	*26*.*4*
With cardiac rehabilitation*	2,478	*65*.*8*
Ischemic complication** or death	303	*8.0*
Ischemic complication	194	*5*.*1*
Stent thrombosis	21	*0*.*6*
Myocardial infarction	50	*1*.*3*
Non-hemorrhagic stroke	17	*0*.*5*
Unplanned revascularization	157	*4*.*2*
Target lesion revascularization	32	*0*.*8*
Death	130	*3*.*5*
Hemorrhagic complication	73	*1.9*

The italic values in % (percentage in line): number of patients with the characteristic divided by the total number of patients included.

* International Classification of Diseases (ICD)-10 diagnosis code Z50.0 “Cardiac rehabilitation” as the main care registered.

** Stent thrombosis, myocardial infarction, non-hemorrhagic stroke, unplanned revascularization, target lesion revascularization.

Over the 1-year follow-up, 303 patients (8.0%) had a pejorative outcome: ischemic complication (*n* = 194; 5.1%) and/or death (*n* = 130; 3.5%) (Table 2). This proportion varied according to patients' characteristics and management (Table 3).

**Table 3:** Ischemic complication and/or death at 1 year after STEMI according to their characteristics and management—the STOP-SCA+ study.

The STOP-SCA+ study: patients' characteristics	Total	Among whom, ischemic complication and/or death (*n* = 303)	Univariable analysis *p*-value (log-rank)
	*n*	% (*in line*)	
All patients	3,768	*8*.*0*	
Age ≥65 years old	1,601	*10*.*7*	<0.01
Women	936	*7*.*7*	0.67
Comorbidities			
Obesity (BMI ≥ 30 kg/m2)	789	*7*.*2*	0.34
Renal impairment	52	*26*.*9*	<0.01
High blood pressure	1,510	*10*.*1*	<0.01
Diabetes	519	*14*.*6*	<0.01
Smoking (current or past)	2,006	*7*.*7*	0.33
Medical history			
TCA/myocardial infarction/coronary bypass	465	*12*.*9*	<0.01
Stroke	94	*13*.*8*	0.03
Peripheral vascular disease	99	*19*.*2*	<0.01
Family history of coronary disease	797	*7*.*5*	0.56
Killip = 3 or 4	132	*17*.*4*	<0.01
Reperfusion procedure			0.83
Primary angioplasty	3,171	*8*.*1*	
Isolated fibrinolysis	54	*11*.*1*	
Secondary angioplasty	272	*7*.*7*	
Isolated coronary angiography	271	*7*.*4*	
LVEF at discharge <40%*	665	*12*.*2*	<0.01
No cardiac rehabilitation	1,290	*12*.*3*	<0.01

The italic values in % (percentage in line): number of patients with the characteristic divided by the total number of patients included.

BMI, body mass index; LVEF, left ventricular ejection fraction; MI, myocardial infarction; TCA, transluminal coronary angioplasty.

* Missing data: *n* = 362.

**Figure 2:**
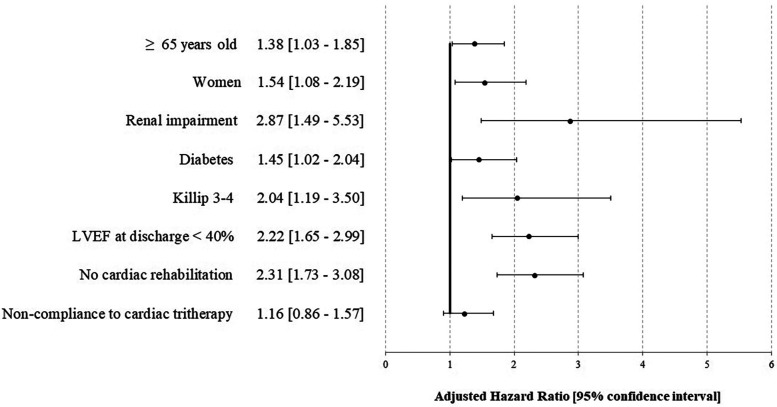
Factors associated with an ischemic complication and/or death at 1 year after STEMI—the STOP-SCA+ study. LVEF, left ventricular ejection fraction.

**Figure 3:**
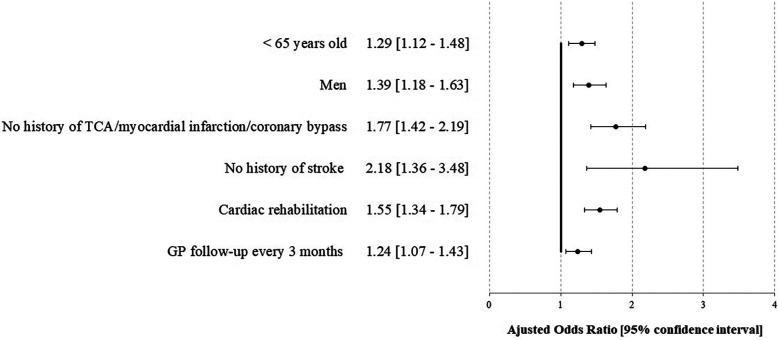
Factors associated with compliance for the cardiac tri-therapy (PDC ≥80%) at 1 year after STEMI—the STOP-SCA+ study. TCA, transluminal coronary angioplasty; GP, general practitioner*.*

In multivariable analyses, compliance was not associated with complications over the year [HR 1.16 (0.86–1.57)], while the absence of cardiac rehabilitation [2.31 (1.73–3.08)] was associated, as well as female sex [1.54 (1.08–2.19)], renal impairment [2.87 (1.49–5.53)], initial clinical presentation [pejorative Killip 2.04 (1.19–3.50)], and LVEF <40% at discharge 2.22 (1.65–2.99) (Figure 2, Supplementary Figures S5 and S7). No interaction was found between cardiac rehabilitation and cardiac tri-therapy compliance. Social deprivation was not associated with pejorative outcomes in univariable analysis (results not reported), thus not included in the multivariable analysis. Studying the factors associated with cardiac tri-therapy compliance (PDC ≥80%), cardiac rehabilitation was a contributive factor [OR = 1.55 (1.34–1.79)] (Figure 3, Supplementary Figure S6), along with a younger age, being a male, and the absence of a medical history of stroke or myocardial infarction (Figure 3).

## Discussion

### Main findings

This study showed that few complications nor deaths occurred in the year following a STEMI. Only 8% of the patients had poor outcomes, lower than reported elsewhere, although comparisons were limited due to different inclusion criteria and outcomes (21–23). Pejorative outcomes were mainly related to patients' pre-existing conditions (age ≥65 years old, female sex, renal impairment, and diabetes) and the initial severity of the STEMI (Killip 3–4 and LVEF at discharge <40%) and also associated with the absence of cardiac rehabilitation (adjusted HR 2.3), whereas the effect of compliance to cardiac tri-therapy could not be demonstrated through the sensitivity analyses. Our results aligned with those of the FAST-MI registry which showed a significant reduction in 1-year mortality risk in a French cohort of patients admitted to cardiac rehabilitation after an acute myocardial infarction (24, 25). Here, we found that almost all STEMI patients were prescribed at least once aspirin (98% of patients), a P2Y12 inhibitor (90%), or a statin (97%), resulting in 89% with at least one tri-therapy prescription, but only one half of them had a PDC ≥ 80%. This definition of compliance was very stringent; however, the initial results showed that the absence of medicine uptake once in a while had no pejorative consequences. It might have been interesting to consider different thresholds of compliance in sensitivity analysis. We also emphasized the importance of considering medical compliance both as a static parameter, such as the PDC over a defined period, and as a dynamic parameter, such as a time-dependent variable, regardless of the total duration of deliveries. Our results provided insights into a field where no consensus exists regarding the duration of DAPT or statin therapy following STEMI. According to the 2017 European Society of Cardiology (ESC) guidelines, “multiple studies have shown that shortening DAPT to 6 months, compared with 12 months or longer, reduces the risk of major bleeding complications, with no apparent trade-off in ischaemic events,” while “two major studies have shown the benefit towards reduction of non-fatal ischaemic events in patients receiving longer than 12 months of DAPT.” The guidelines conclude that “no formal recommendations are possible for the use of clopidogrel or prasugrel beyond 1 year” (26). Regarding the long-term prescription of statins, although strongly recommended, the ESC guidelines do not specify a duration. Instead, they state that “lipids should be re-evaluated 4–6 weeks after the [acute coronary syndrome] to determine whether the target levels have been reached and regarding safety issues; the lipid lowering therapy can then be adjusted accordingly.” Long-term follow-up of patients enrolled in clinical trials testing lipid-lowering therapy in high-risk patients supports a favorable risk–benefit ratio for prolonged LDL-lowering therapy (27). Other post-STEMI treatments recommended in the BASI were not considered for this study (4, 13), due to numerous posology adaptations over time, not allowing to reliably assess compliance.

Furthermore, our study took into account post-STEMI cardiac rehabilitation through the SNDS database, which appeared to be a major factor in improving the 1-year outcome, as previously suggested, although a strong causal model remains to be built (28, 29). Our results were particularly encouraging, as two-thirds of the patients benefited from cardiac rehabilitation, which was higher than previous data, where about a third of coronary patients in Europe receive any form of cardiac rehabilitation and only 25% in the USA (19, 24). However, it was recently shown that patients with STEMI were admitted to cardiac rehabilitation twice as often as patients without ST-segment elevation (30). In France, Puymirat et al. (24) demonstrated the benefits of cardiac rehabilitation after admission for acute myocardial infarction with a risk reduction of 1-year mortality.

### Study limitations

First, an observational design could not provide such causal inferences as a randomized control trial (RCT) (31). A way of emulating an RCT is to perform a propensity score-based study. However, some studies highlighted that using a propensity score does not change the odds ratios as compared with logistic or Cox regression models (32, 33). To overcome imbalances in baseline characteristics, we performed sensitivity analyses, including the inverse probability of treatment weighting (IPTW), giving discordant results, and not allowing us to demonstrate an association between compliance and pejorative outcomes. It would be interesting to repeat the study in a few years when a higher number of events is reached. Moreover, we could not eliminate a potential immortal time bias, even if we showed that the median time from discharge to rehabilitation admission was not different between patients with or without any pejorative outcomes.

Moreover, the definition of compliance through the SNDS medico-administrative database could be challenging (18, 34). The SNDS contains the dispensations and not the administration, and delivery discontinuations cannot be identified, resulting in a possible misestimation of compliance, underestimation for patients for whom the delivery was stopped due to health or tolerance issues, whereas overestimation of compliance in case of self-discontinuation by patients who do not feel the usefulness of the treatment in the absence of symptoms (treatment delivered but not taken). Moreover, the SNDS did not take into account a number of other confounders that could affect both medication compliance and outcomes, such as environmental factors. Social deprivation was considered through the French deprivation index “FDep” (20), and no interaction was found with cardiac tri-therapy compliance. Given these limitations, the inconclusive results about compliance and pejorative outcomes could not allow any definitive conclusions. Further studies are needed to drop the concerns, and larger populations that could be available in real-world registries, such as France PCI, the national extension of the CRAC registry, could provide valuable insights.

The CRAC registry has also some limitations: the inclusion criterion is limited to patients admitted to ICCs. Thus, STEMI patients without any invasive procedure, even if rare, are not analyzed, resulting in a selection bias. However, previous studies estimated that hospitalized STEMI patients without coronary angiography corresponded to <5% in France (19, 35).

### Strengths

The originality and interest of our study are multiple. First, our study was the first to date matching two complementary databases: the clinico-biological data from the CRAC registry (a subgroup of France PCI), including a large STEMI population of nearly 4,000 individuals over 5 years, and the medico-administrative data of the SNDS, allowing to increase the completeness and accuracy of the results. The SNDS proved its epidemiological interest, allowing us to catch complete and large-scale data, especially after discharge in outpatient care, which usually requires an onsite follow-up led by local research technicians, impacting cost and resulting in an underestimation of the results due to lost to follow-up/dead patients. Moreover, one of the major strengths of this database was the possibility to perform analyses based on real-life data allowing us to study the real care continuum. The regional registry, initially covering five ICCs is now extended to over 89 ICCs across the country in the France PCI initiative. As the SNDS is part of the French health data hub (https://www.francepci.fr), this will enable the extension of our findings to a national level in the near future. Eventually, the matching was reliable and efficient and could provide an estimation of the operational resources required, assessed in real-life settings. All these elements assess the generalizability and robustness of the findings.

## Conclusions

This study showed that patients admitted for emergency revascularization at the acute phase of a STEMI mainly had favorable outcomes at 1 year, with few complications or death. Pejorative outcomes appeared to be related to patients' characteristics, initial clinical presentation, and access to rehabilitation care, whereas no impact could be observed for non-compliance. Matching two complementary clinical and medico-administrative databases proved to be reliable for assessing outcomes on a large scale. This permanent and multicentric database with systematic long-term follow-up in ICC provides excellent quality data and immediate feedback at a large scale, without high cost nor changing usual practice due to a fully integrated electronic medical report. This large reliable database could soon provide care quality improvement linked to benchmarking and promote robust studies.

## Data Availability

The data analyzed in this study is subject to the following licenses/restrictions: The data underlying this article cannot be shared publicly due to privacy and legal reasons, according to the decision from the French Data Protection Board [Commission Nationale de l'Informatique et des Libertés (CNIL)], decision DR-2021-025 (Jan. 28th, 2021). Requests to access these datasets should be directed to leslie.guillon@univ-tours.fr.
