# Correction: Impact of cardiac rehabilitation and treatment compliance after ST-segment elevation myocardial infarction (STEMI) in France, the STOP SCA+ study

**DOI:** 10.3389/fcvm.2025.1656799

**Published:** 2025-09-11

**Authors:** Emeline Laurent, Lucile Godillon, Marc-Florent Tassi, Pierre Marcollet, Stéphan Chassaing, Marie Decomis, Julien Bezin, Christophe Laure, Denis Angoulvant, Grégoire Range, Leslie Grammatico-Guillon

**Affiliations:** ^1^ Public Health Unit, Epidemiology, Teaching Hospital of Tours, Tours, France; ^2^ Research Unit EA 7505 “Education, Ethics and Health”, University of Tours, Tours, France; ^3^ Faculty of Pharmacy, University of Tours, Tours, France; ^4^ Cardiology Department, CH Bourges, Bourges, France; ^5^ Cardiology Department, Private Hospital NCT+, Tours, France; ^6^ Cardiology Department, Private Hospital Oréliance, Orléans, France; ^7^ Clinical Pharmacology Unit, University of Bordeaux, INSERM, BPH, Team AHeaD, Bordeaux, France; ^8^ Cardiology Department, Les Hôpitaux de Chartres, Chartres, France; ^9^ Cardiology Department, Teaching Hospital of Tours, Tours, France; ^10^ Faculty of Medicine, University of Tours, Tours, France

**Keywords:** myocardial infarction (MI), cardiac rehabilitation, compliance, outcome, probabilistic matching

The figures were in the wrong order in the PDF and HTML version of this paper. Figure 2 should have been figure 3 and vice versa. The order has now been corrected.

**Figure 2:**
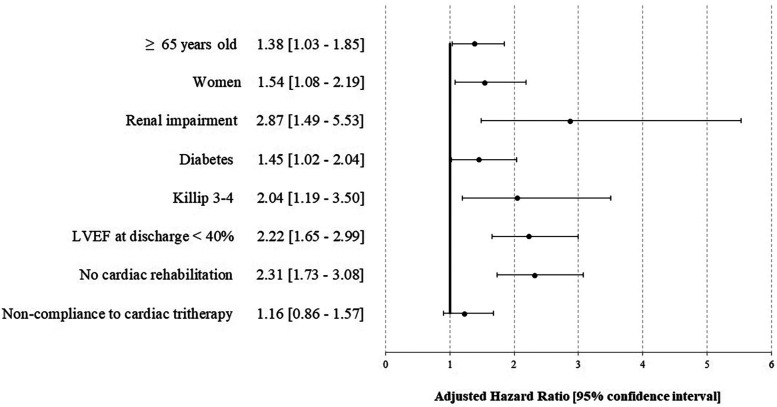
Factors associated with an ischemic complication and/or death at 1 year after STEMI—the STOP—SCA+ study. LVEF, left ventricular ejection fraction.

**Figure 3:**
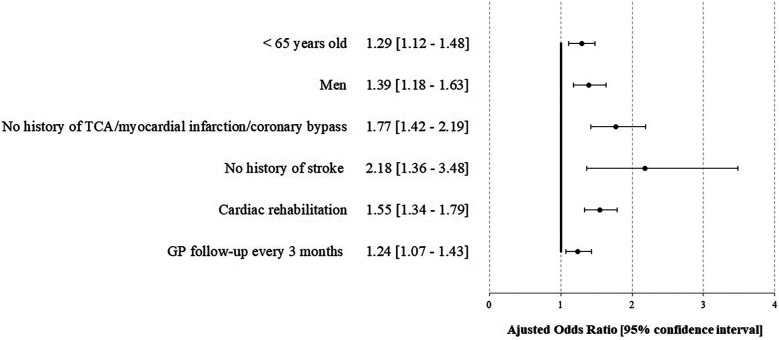
Factors associated with compliance for the cardiac tri-therapy (PDC ≥80%) at 1 year after STEMI-the STOP-SCA+ study. TCA, transluminal coronary angioplasty; GP, general practitioner.

The original version of this article has been updated.

