# Supplementary material for: Impact of cardiac rehabilitation and treatment compliance after ST-segment elevation myocardial infarction (STEMI) in France, the STOP SCA+ study
Source: Front Cardiovasc Med. 2025 Jun 12;12:1484401. doi: 10.3389/fcvm.2025.1484401 (PMC12198249; doi:10.3389/fcvm.2025.1484401)
Supplement: Supplementary file 1 [file Datasheet1.docx]

**Supplementary material**

Impact of cardiac rehabilitation and treatment compliance after ST-segment elevation myocardial infarction (STEMI) in France, the STOP SCA+ study

**S1. Probabilistic matching between the CRAC registry and SNDS data – The STOP-SCA+ study**

The probabilistic matching between the CRAC Registry and the SNDS was performed according to:

• the sex of the patient

• the patient's age at the time of the stay during which the interventional cardiology procedure was performed.

• the administrative identifier (FINESS) of the centre that performed the interventional cardiology procedure

• the date of the intervention (angioplasty or coronary angiography)

• the admission and discharge dates from the stay during which the interventional cardiology procedure was performed

In the absence of a variable for the patient's place of residence in the CRAC Registry (only the place of occurrence of the chest pain is registered), no matching could be carried out on the geographical criterion.

A seven-step probabilistic matching was realised as described below:

**Table S1.1. Probabilistic matching steps between the CRAC registry and SNDS data – The STOP-SCA+ study**

|  | ICC | Sex | Age | Intervention date | Admission date | Discharge date |
| --- | --- | --- | --- | --- | --- | --- |
| Step 1 | • | • | • | • | • | • |
| Step 2 | • | • | • | • | • |  |
| Step 3 | • | • | • | • |  | • |
| Step 4 | • | • | • |  | • | • |
| Step 5 | • | • | ± one year | • | • | • |
| Step 6 | • | • | • | ± one day |  |  |
| Step 7^*^ |  | • | • | ± one day | |  |

** Inter-hospital Provision only, i.e. outsourcing coronary angiography/PCI to another hospital*

*Abreviations, ICC: Interventional Cardiac Centre identifier; PCI: Percutaneous Intervention*

**Table S1.2. Matched patients and performance parameters for each matching step - The STOP-SCA+ study**

|  | Matched patients | TP | TN | FP | FN | Sensitivity | Specificity | Positive predictive value | Negative predictive value |
| --- | --- | --- | --- | --- | --- | --- | --- | --- | --- |
| Step 1 | 3523 | 3510 | 656 | 4 | 9 | 99,7% | 99,4% | 99,9% | 98,6% |
| Step 2 | 185 | 183 | 471 | 0 | 2 | 98,9% | 100,0% | 100,0% | 100,0% |
| Step 3 | 52 | 51 | 419 | 0 | 1 | 98,0% | 100,0% | 100,0% | 100,0% |
| Step 4 | 73 | 73 | 346 | 0 | 0 | 100,0% | 100,0% | 100,0% | 100,0% |
| Step 5 | 109 | 108 | 237 | 0 | 1 | 99,0% | 100,0% | 100,0% | 100,0% |
| Step 6 | 33 | 27 | 204 | 0 | 6 | 81,8% | 100,0% | 100,0% | 97,1% |
| Step 7 | 48 | 42 | 156 | 0 | 6 | 87,5% | 100,0% | 100,0% | 96,3% |

TP, True Positive: one CRAC patient merging with one hospital stay (PMSI SNDS).

TN, True Negative: one CRAC patient without any hospital stay retrieved.

FP, False Positive or "collision": different CRAC patients merging with one same hospital stay.

FN False Negative or "duplicate": one same CRAC patient merging with different hospital stays.

At each step, false-positive and false-negative cases were checked and manually corrected, thus becoming true positive cases. For clarity purpose, these cases were reported in the table with their original classification.

The probabilistic matching process, combined with the afterward selection of patients meeting the inclusion criteria, resulted in the flow-chart below:

**Figure S1.3.** **Flow-chart – The STOP-SCA+ study**

**S2. Detailed description of cardiac drug deliveries at one year after a ST elevation myocardial infarction STEMI – The STOP-SCA+ study**

| At least one delivery at one year  - the *STOP-SCA+* study | | **Included patients** | |
| --- | --- | --- | --- |
|  |  | **n** | **%** |
| **Total number of patients** | | **3,768** | ***100.0*** |
| Aspirin | | 3,676 | *97.6* |
| Other antiplatelet therapy | | 3,417 | *90.7* |
|  | Clopidogrel | 611 | *16.2* |
|  | Prasugrel | 195 | *5.2* |
|  | Ticagrelor | 2,843 | *75.5* |
| Statin therapy | | 3,660 | *97.1* |
| Other lipid lowering therapy | | 254 | *6.7* |
| Angiotensin-converting enzyme Inhibitor | | 3,080 | *81.7* |
| Angiotensin II receptor blocker (ARB) / diuretic therapy | | 494 | *13.1* |
| Beta-blocker | | 3,568 | *94.7* |
| Anticoagulant therapy | | 534 | *14.2* |
| Nitrate | | 2,167 | *57.5* |
| No cardiac treatment | | 7 | *0.2* |

**S3. Detailed description of cardiac drug deliveries at one year after a ST elevation myocardial infarction STEMI – The STOP-SCA+ study**

|  |  | ≥1 delivery (patients) |  | Proportion of days covered (PDC) | | | | | | | | | | |
| --- | --- | --- | --- | --- | --- | --- | --- | --- | --- | --- | --- | --- | --- | --- |
|  |  |  |  | 0%  (not prescribed or never delivered) | |  | [1%-50%[ | |  | [50%-80%[ | |  | ≥ 80% | |
|  |  | n |  | n | % |  | n | % |  | n | % |  | n | % |
| Aspirin (A) |  | 3,676 |  | 92 | 2.4% |  | 186 | 4.9% |  | 481 | 12.8% |  | 3,009 | 79.9% |
| Other antiplatelet therapy (B) |  | 3,417 |  | 351 | 9.3% |  | 431 | 11.4% |  | 453 | 12.0% |  | 2,533 | 67.2% |
| Statin therapy (C) |  | 3,660 |  | 108 | 2.9% |  | 216 | 5.7% |  | 493 | 13.1% |  | 2,951 | 78.3% |
| Dual antiplatelet therapy : A + B |  | 3,402 |  | 366 | 9.7% |  | 474 | 12.6% |  | 597 | 15.8% |  | 2,331 | 61.9% |
| Cardiac tri-therapy: A + B + C |  | 3,362 |  | 406 | 10.8% |  | 582 | 15.4% |  | 785 | 20.8% |  | 1,995 | 52.9% |

**S4. Kaplan–Meier curves showing the cumulative probabilities of survival without ischaemic complications, up to one year after STEMI – The STOP-SCA+ study**

**
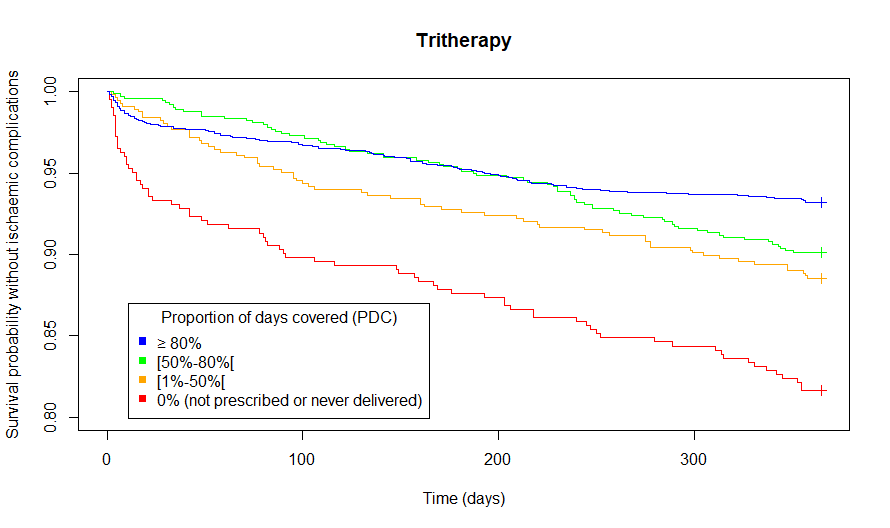
**

**S5. Factors associated with an ischaemic complication and/or death at one year after STEMI – the STOP-SCA+ study.**

|  |  |  | **Total** |  | **Among whom, ischaemic complication and/or death (n=303)** |  | univariable **p-value  (log-rank)** |  | **Multivariable analysis  (Cox model) (n=3,319)** | |
| --- | --- | --- | --- | --- | --- | --- | --- | --- | --- | --- |
|  |  |  | **n** |  | ***%*** *(in line)* |  |  |  | **HR** | **95%CI** |
| **All patients** | |  | 3,768 |  | *8.0* |  |  |  |  |  |
| **Age ≥65 years old** | |  | 1,601 |  | *10.7* |  | <0.01 |  | 1,38 | [1.03-1.85] |
| **Women** | |  | 936 |  | *7.7* |  | 0.67 |  | 1.54 | [1.07-2.22] |
| **Comorbidities** | |  |  |  |  |  |  |  |  |  |
|  | Obesity (BMI ≥ 30kg/m2) |  | 789 |  | *7.2* |  | 0.34 |  | - |  |
|  | Renal impairment |  | 52 |  | *26.9* |  | <0.01 |  | 2,87 | [1.49-5,53] |
|  | High blood pressure |  | 1,510 |  | *10.1* |  | <0.01 |  | - |  |
|  | Diabetes |  | 519 |  | *14.6* |  | <0.01 |  | 1,45 | [1.02-2.04] |
|  | Smoking (current or past) |  | 2,006 |  | *7.7* |  | 0.33 |  | - |  |
| **Medical history** | |  |  |  |  |  |  |  |  |  |
|  | TCA/myocardial infarction/coronary bypass |  | 465 |  | *12.9* |  | <0.01 |  | - |  |
|  | Stroke |  | 94 |  | *13.8* |  | 0.03 |  | - |  |
|  | Peripheral vascular disease |  | 99 |  | *19.2* |  | <0.01 |  | - |  |
|  | Family history of coronary disease |  | 797 |  | *7.5* |  | 0.56 |  | - |  |
| **Killip = 3 or 4** | |  | 132 |  | *17.4* |  | <0.01 |  | 2,04 | [1.19-3.50] |
| **Reperfusion procedure** | |  |  |  |  |  | 0.83 |  | - |  |
|  | Primary angioplasty |  | 3,171 |  | *8.1* |  |  |  |  |  |
|  | Isolated fibrinolysis |  | 54 |  | *11.1* |  |  |  |  |  |
|  | Secondary angioplasty |  | 272 |  | *7.7* |  |  |  |  |  |
|  | Isolated coronary angiography |  | 271 |  | *7.4* |  |  |  |  |  |
| **LVEF at discharge <40%*** | |  | 665 |  | *12.2* |  | <0.01 |  | 2,22 | [1.65-2.99] |
| **No cardiac rehabilitation**  (time-dependent variable) | |  |  |  |  |  |  |  | 2,31 | [1.73-3.08] |
| **Non-compliance to cardiac tri-therapy**  (time-dependent variable) | |  |  |  |  |  |  |  | 1,16 | [0.86-1.57] |
| * missing data: n=362 | |  |  |  |  |  |  |  |  |  |
| BMI: Body Mass Index; LVEF: Left Ventricular Ejection Fraction; MI: Myocardial Infarction; TCA: Transluminal Coronary Angioplasty | | | | | | | | | | |
| HR: Hazard Ratio; 95%CI: 95% Confidence Interval | |  |  |  |  |  |  |  |  |  |

**S6. Factors associated with cardiac tri-therapy compliance (percentage of days covered ≥80%) at one year after STEMI – the STOP-SCA+ study.**

| STEMI patients from the STOP-SCA+ study, except patients with at least one major adverse cardiac and/or cerebral event during the year | |  | **Total** |  | **Among whom, PDC ≥80% for tri-therapy** |  | **Univariable  p-value** |  | **Multivariable analysis** | | | | |  |
| --- | --- | --- | --- | --- | --- | --- | --- | --- | --- | --- | --- | --- | --- | --- |
|  |  |  | **n** |  | ***%*** *(in line)* |  |  |  | **OR** | | **95%CI** | | |  |
| **All patients** | |  | 3,506 |  | *53.4* |  |  |  |  | |  | | |  |
| **Age <65 years old** | |  | 2,040 |  | *57.8* |  | <0.01 |  | 1.29 | | 1.12-1.48 | | |  |
| **Men** | |  | 2,630 |  | *55.8* |  | <0.01 |  | 1.39 | | 1.18-1.63 | | |  |
| **Medical history** | |  |  |  |  |  |  |  |  | |  | | |  |
|  | No TCA/myocardial infarction/coronary bypass |  | 3,090 |  | *55.3* |  | <0.01 |  | 1.77 | | 1.42-2.19 | | |  |
|  | No stroke |  | 3,416 |  | *54.0* |  | <0.01 |  | 2.18 | | 1.36-3.48 | | |  |
|  | No peripheral vascular disease |  | 3,413 |  | *53.7* |  | 0.02 |  | - | |  | | |  |
| **Cardiac rehabilitation** | |  | 2,320 |  | *58.2* |  | <0.01 |  | 1.55 | | 1.34-1.79 | | |  |
| **Less than four cardiac drugs  (in addition to the tri-therapy)** | |  | 2,943 |  | *54.8* |  | <0.01 |  | - | |  | | |  |
| **≥ 1 visit with a GP every 3 months** | |  | 2,328 |  | *55.1* |  | 0.01 |  | 1.24 | | 1.07-1.43 | | |  |
| **≥ 1 visit with a cardiologist every 6 months** | |  | 1,107 |  | *52.7* |  | 0.54 |  | - | |  | | |  |
| **Potential access to a GP*** | |  |  |  |  |  | 0.15 |  | - | |  | | |  |
|  | Low (<2) |  | 218 |  | *59.6* |  |  |  |  | |  | | |  |
|  | Moderate ([2-4[) |  | 1,217 |  | *52.8* |  |  |  |  | |  | | |  |
|  | High (≥4) |  | 922 |  | *53.0* |  |  |  |  | |  | | |  |
| **Less-favoured area**** | |  | 1,817 |  | *53.6* |  | 0.27 |  | - | |  | | |  |
| * https://www.irdes.fr/Publications/2012/Qes174.pdf  ** according to a deprivation score (Fdep) for each French area, divided in 5 quintiles; the two last quintiles were considered as less-favoured | | | | | | | | | | | | | | |
| GP: generalist practitioner; PDC: Percentage of Days Covered; TCA: Transluminal Coronary Angioplasty | | | | | | | | | | | | | | |
| OR: Odds Ratio; 95%CI: 95% Confidence Interval | |  |  |  |  |  |  | | |  | |  |  | |

**S7.** **Sensitivy analyses to study association between cardiac tri-therapy and an ischaemic complication and/or death at one year after a STEMI**

|  |  | **Without adjustment** | | **Cox model with covariate* adjustment** | | **Cox model with propensity score adjustment** | | | **Cox model with weighting observations by IPTW**** | | | |
| --- | --- | --- | --- | --- | --- | --- | --- | --- | --- | --- | --- | --- |
|  |  | **(n=3,319)** | | **(n=3,319)** | | **(n=3,319)** | | | **(n=3,319)** | | | |
|  |  | **HR** | **95%CI** | **HR** | **95%CI** | **HR** | **95%CI** | | **HR** | | **95%CI** | |
| **Non-compliance to cardiac tri-therapy** | | 1.43 | [1.07-1.92] | 1.16 | [0.86-1.57] | 1.19 | [0.88-1.61] | | 1.30 | | [1.05-1.60] | |
| (time-dependent variable) | |  |  |  |  |  |  |  |  |  |  |  |
| ** age, sex, renal impairment, diabetes, LVEF, Killip, cardiac rehabilitation* | | | | | | | |  | |  | |  |
| *** Inverse probability of treatment weighting* | | | |  |  |  |  | |  | |  | |
